# Differential Expression of Endogenous Retroviruses and Inflammatory Mediators in Female and Male Offspring in a Mouse Model of Maternal Immune Activation

**DOI:** 10.3390/ijms232213930

**Published:** 2022-11-11

**Authors:** Chiara Cipriani, Anna Maria Tartaglione, Martina Giudice, Erica D’Avorio, Vita Petrone, Nicola Toschi, Flavia Chiarotti, Martino Tony Miele, Gemma Calamandrei, Enrico Garaci, Claudia Matteucci, Paola Sinibaldi-Vallebona, Laura Ricceri, Emanuela Balestrieri

**Affiliations:** 1 Department of Experimental Medicine, University of Rome Tor Vergata, Via Montpellier 1, 00133 Rome, Italy; 2 Center for Behavioral Sciences and Mental Health, Istituto Superiore di Sanità (ISS), 00161 Rome, Italy; 3 Department of Biomedicine and Prevention, Tor Vergata University of Rome, 00133 Rome, Italy; 4 Martinos Center for Biomedical Imaging, Harvard Medical School, Boston, MA 02115, USA; 5 University San Raffaele, 00166 Rome, Italy; 6 IRCCS San Raffaele Pisana, 00163 Rome, Italy; 7 Institute of Translational Pharmacology, National Research Council, 00133 Rome, Italy

**Keywords:** endogenous retroviruses (ERVs), autism spectrum disorder, social behavior, Poly I:C, cytokines, neuroinflammation, maternal immune activation (MIA), gene expression

## Abstract

Maternal infections during pregnancy and the consequent maternal immune activation (MIA) are the major risk factors for autism spectrum disorder (ASD). Epidemiological evidence is corroborated by the preclinical models in which MIA leads to ASD-like behavioral abnormalities and altered neuroinflammatory profiles, with an increase in pro-inflammatory cytokines and microglial markers. In addition to neuroinflammatory response, an abnormal expression of endogenous retroviruses (ERVs) has been identified in neurodevelopmental disorders and have been found to correlate with disease severity. Our aim was to evaluate the transcriptional profile of several ERV families, ERV-related genes, and inflammatory mediators (by RT real-time PCR) in mouse offspring of both sexes, prenatally exposed to polyinosinic:polycytidylic acid (Poly I:C), a synthetic double-stranded RNA molecule targeting TLR-3 that mimics viral maternal infection during pregnancy. We found that prenatal exposure to Poly I:C deregulated the expression of some ERVs and ERV-related genes both in the prefrontal cortex (PFC) and hippocampus, while no changes were detected in the blood. Interestingly, sex-related differences in the expression levels of some ERVs, ERV-related genes, and inflammatory mediators that were higher in females than in males emerged only in PFC. Our findings support the tissue specificity of ERV and ERV-related transcriptional profiles in MIA mice.

## 1. Introduction

Maternal infections during pregnancy are one of the major environmental risk factors for neuropsychiatric conditions, such as schizophrenia, bipolar disorders, and autism spectrum disorder (ASD), occurring in the offspring [1,2,3]. Clinical and preclinical evidence attributes to maternal immune activation (MIA) an important role in neurodevelopmental deviations caused by maternal infection. Indeed, MIA is primarily triggered by infections and autoimmune diseases in the mother, resulting in increased levels of cytokines and chemokines that cross the placental and blood-brain barriers, affecting neural development of the fetus [4,5]. Associations between ASD and prenatal infection with specific pathogens have been repeatedly reported [6,7], with an increased risk of ASD related to the severity of maternal infection. Epidemiological evidence is corroborated by the preclinical models in which prenatal immunostimulant injections (i.e., Toll-like receptor (TLR) agonists and cytokines) lead to ASD-like neurobiological and behavioral abnormalities in offspring [8,9]. Together with long-term behavioral alterations, MIA mouse offspring exhibit an altered neuroinflammatory profile, with an increase in pro-inflammatory cytokines and microglial markers [10,11,12,13]. In addition to the neuroinflammatory response, an abnormal expression of endogenous retroviruses (ERVs) has been identified in neurodevelopmental disorders including ASD and has been correlated with disease severity and pharmacological treatment [14,15,16,17,18]. ERVs are derived from their exogenous retroviral counterparts by a process of germline infection and proliferation within the host genome [19,20]. The majority of ERVs lack function due to negative selective pressures and mutations, which occurred during evolution [21]. Some ERVs have been co-opted for physiological functions [22,23], while their activation has been frequently associated with pathological conditions with a complex and multifactorial etiology in which HERVs seem to represent the epigenetic interface between genetics, the environment, and the immune system [14,24,25]. ERV sequences are highly represented within the mammalian genomes as they account for 5% to 10% of the genetic material [26,27]. In the mouse genome, several groups of retro-transpositionally active ERVs are the cause of the most reported insertional mutations and they are recognized as drivers of germline transcriptome and as cofactors in different crucial phases of embryogenesis [28,29,30]. Interestingly, we previously reported that the inbred mouse strain BTBR T+Itpr3tf/J (BTBR), considered a model of idiopathic autism, and outbred mice prenatally exposed to valproic acid (VPA), showed higher expression levels of ERVs from intrauterine life up to adulthood compared to relative controls. The aberrant expression of some ERV families positively correlated with expression levels of pro-inflammatory cytokines and TLR-3 and TLR-4 in embryos and brain tissues, supporting the interplay between ERVs and the immune response [31]. 

The aim of the present study was to evaluate the transcriptional profile of several ERV families, ERV-related genes, and inflammatory mediators in brain and blood samples from MIA mouse offspring of both sexes, prenatally exposed to polyinosinic:polycytidylic acid (Poly I:C), a synthetic double-stranded RNA molecule targeting TLR-3 that mimics viral maternal infection during pregnancy [32]. 

## 2. Results

### 2.1. Poly I:C Mice Showed Tissue-Specific Expression of Several ERVs, ERV-Related Genes, and Inflammatory Mediators

**Figure 1:**
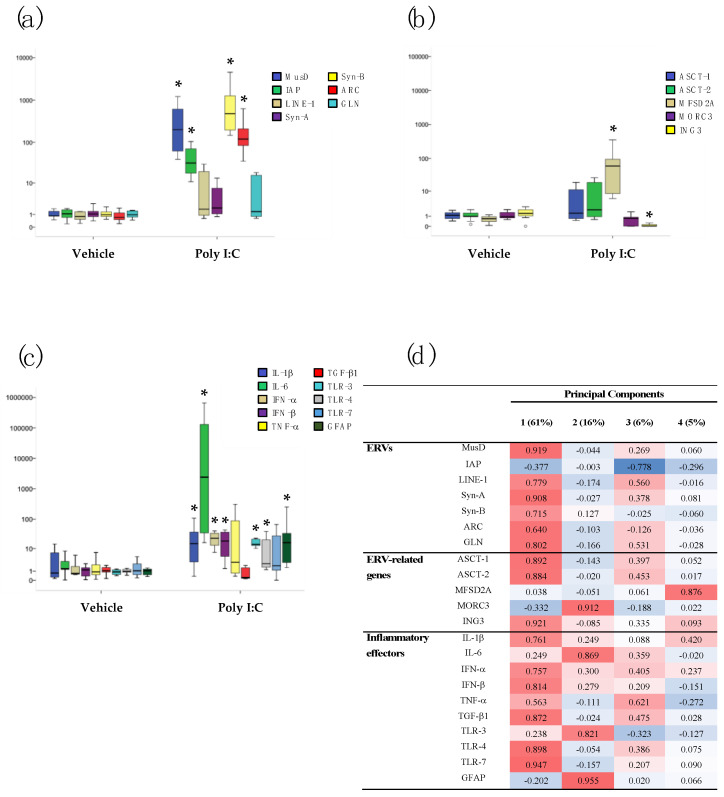
Relative expression of several ERV families (**a**), ERV-related genes (**b**), and inflammatory mediators (**c**) in prefrontal cortex (PFC) of Vehicle and Poly I:C mice (both sexes pooled). Data are represented as box plots with median value (black horizontal line) and first/third interquartile range. * *p* < 0.05 (Poly I:C vs. Vehicle). PCA and hierarchical clustering of the transcriptional levels of the same genes in PFC samples of Poly I:C mice (**d**). Percentage of total variance explained by the component is reported in the head columns between parentheses; red indicates positive association, blue indicates negative associations, and color intensity refers to association strength.

**Figure 2:**
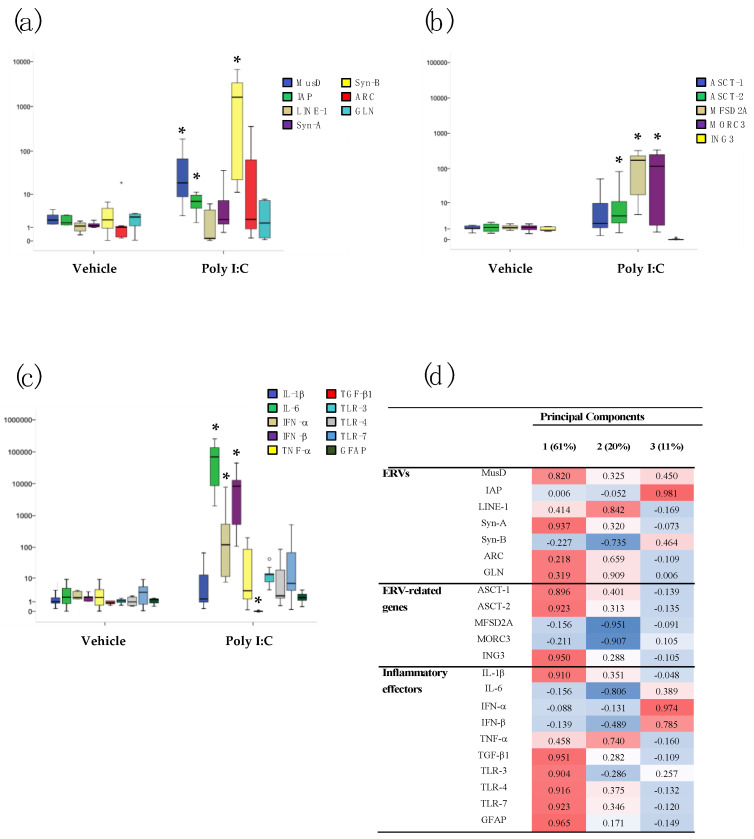
Relative expression of several ERV families (**a**), ERV-related genes (**b**), and inflammatory mediators (**c**) in hippocampus (HP) of Vehicle and Poly I:C mice (both sexes pooled). Data are represented as box plots with median value (black horizontal line) and first/third interquartile range. * *p* < 0.05 (Poly I:C vs. Vehicle). PCA and hierarchical clustering of the transcriptional levels of the same genes in HP samples of Poly I:C mice (**d**). Percentage of total variance explained by the component is reported in the head columns between parentheses; red indicates positive association, blue indicates negative associations, and color intensity refers to association strength.

**Figure 3:**
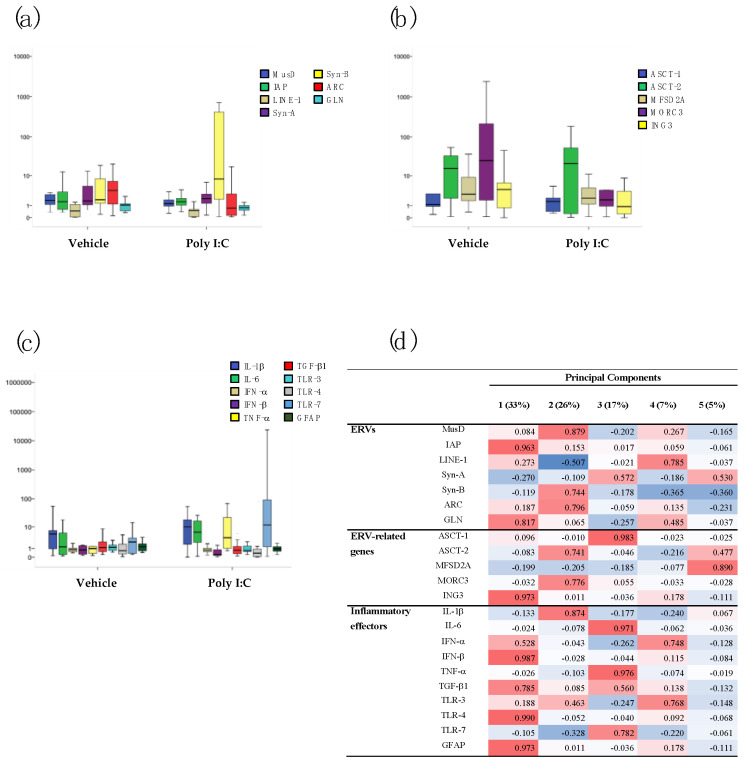
Relative expression of several ERV families (**a**), ERV-related genes (**b**), and inflammatory mediators (**c**) in blood (BL) of Vehicle and Poly I:C mice (both sexes pooled). Data are represented as box plots with median value (black horizontal line) and first/third interquartile range(Poly I:C vs. Vehicle). PCA and hierarchical clustering of the transcriptional levels of the same genes in BL samples of Poly I:C mice (**d**). Percentage of total variance explained by the component is reported in the head columns between parentheses; red indicates positive association, blue indicates negative associations, and color intensity refers to association strength.

The transcriptional activity of several ERV families (MusD, IAP, Syn-A, Syn-B, ARC, and GLN), related genes (ASCT-1, ASCT-2, MFDS2A, MORC3, and ING3), LINE-1, cytokines (IL-1β, IL-6, TNF-α, and TGF-β1), interferons (IFN-α and IFN-β), TLRs (TLR-3, TLR-4, and TLR-7), and a marker of central nervous system damage (GFAP) were analyzed in the brain (prefrontal cortex (PFC) and hippocampus (HP)) and blood (BL) samples of 60-day-old Poly I:C and Vehicle mice of both sexes, by RT real-time PCR assay. The data are represented as box plots in Figure 1, Figure 2 and Figure 3; median values, interquartile ranges, and *p*-values by Mann–Whitney test are reported in Supplementary Table S1.

In the prefrontal cortex, a significant increase in MusD (*p* < 0.001), IAP (*p* < 0.001), Syn-B (*p* < 0.001), ARC (*p* < 0.001) (Figure 1a), and MFSD2A (*p* < 0.001) was observed in Poly I:C mice compared to Vehicle mice along with a reduction in ING3 expression (*p* = 0.001) (Figure 1b). The transcriptional activity of inflammatory mediators, such as IL-1β (*p* = 0.022), IL-6 (*p* < 0.001), IFN-α (*p* < 0.001), IFN-β (*p* < 0.001), TLR-3 (*p* < 0.001), TLR-4 (*p* < 0.001), and GFAP (*p* < 0.001), was also significantly increased in Poly I:C mice compared to the Vehicle group (Figure 1c). 

In the hippocampus, a significant increase in MusD (*p* < 0.001), IAP (*p* < 0.001), and Syn-B (*p* < 0.001) was observed in samples from Poly I:C mice compared to Vehicle mice (Figure 2a). Additionally, ASCT-2 (*p* < 0.001), MFSD2A (*p* < 0.001), and MORC3 (*p* < 0.001) expression was significantly higher in the Poly I:C mice than in Vehicle mice (Figure 2b). Conversely, ING3 expression was significantly lower in Poly I:C group than in the Vehicle mice group (*p* < 0.001) (Figure 2b). Exposure to Poly I:C also increased the transcriptional activity of inflammatory mediators, such as IL-6 (*p* < 0.001), IFN-α (*p* < 0.001), IFN-β (*p* < 0.001), TGF-β1 (*p* < 0.001), and TLR-3 (*p* < 0.001), compared to Vehicle mice (Figure 2c).

In blood samples, no statically significant differences were found (Figure 3).

Principal component analysis (PCA) demonstrated that ERVs are the main carriers of information in the brain areas of Poly I:C-treated mice. Indeed, the PCA performed on the PFC from Poly I:C mice yielded four components, explaining ~88 % of the total variance (Figure 1d): (i) Component 1 carried most of the information (~61% of the total variance) with positive loadings by ERVs (MusD, Syn-A, Syn-B, and GLN), ERV-related genes (ASCT-1 and ASCT-2 and ING3), LINE-1, and inflammatory mediators (IL-1β, IFN-α, IFN-β, TGF-β1, TLR-4, and TLR-7); (ii) Component 2 was positively loaded on MORC3, IL-6, TLR3, and GFAP (~16%). The other two components explained ~6% and ~5% of the variance (respectively) and with main negative and positive loadings by IAP and MFSD2A, respectively. As shown in Figure 2d, the same analysis performed on the HP of Poly I:C mice identified three components explaining ~92 % of the total variance: (i) Component 1, which carried most of the information (~61% of the total variance) on which MusD, Syn-A, ASCT-1, ASCT-2, ING3, IL-1β, TGF-β1, TLR-3, TLR-4, TLR-7, and GFAP loaded positively; (ii) Component 2 (~20%) on which LINE-1, GLN, and TNF-α loaded positively and Syn-B, MFSD2A, MORC3, and IL-6 loaded negatively; and (iii) Component 3 (~11%) on which IAP, IFN-α, and IFN-β loaded positively.

In the case of blood samples from Poly I:C mice, 88% of the variance was explained by five mediators (Figure 3d): (i) Component 1, which explained 33% of the total variance on which IAP, GLN, ING3, IFN-β, TGF-β1, TLR-4, and GFAP loaded positively; (ii) Component 2 (~26%) on which MusD, Syn-B, ARC, ASCT-2, MORC3, and IL-1β loaded positively; and (iii) Component 3 (~17%) on which ASCT-1, IL-6, TNF-α, and TLR-7 loaded positively. The other two components explained ~7% and ~5% of the variance (respectively) and were mainly positively related to LINE-1, IFN-α, TLR-3, and MFSD2A.

### 2.2. Poly I:C Mice Showed Sex-Dependent Differences in the Expression of ERV, ERV-Related Genes, and Inflammatory Mediators in Prefrontal Cortex

The expression of ERVs, ERV-related genes, and inflammatory mediators was evaluated in the two sexes in the PFC, HP, and BL of Poly I:C mice (see Supplementary Tables S2–S4 for median values, interquartile ranges, and *p*-values by the Mann–Whitney test). 

**Figure 4:**
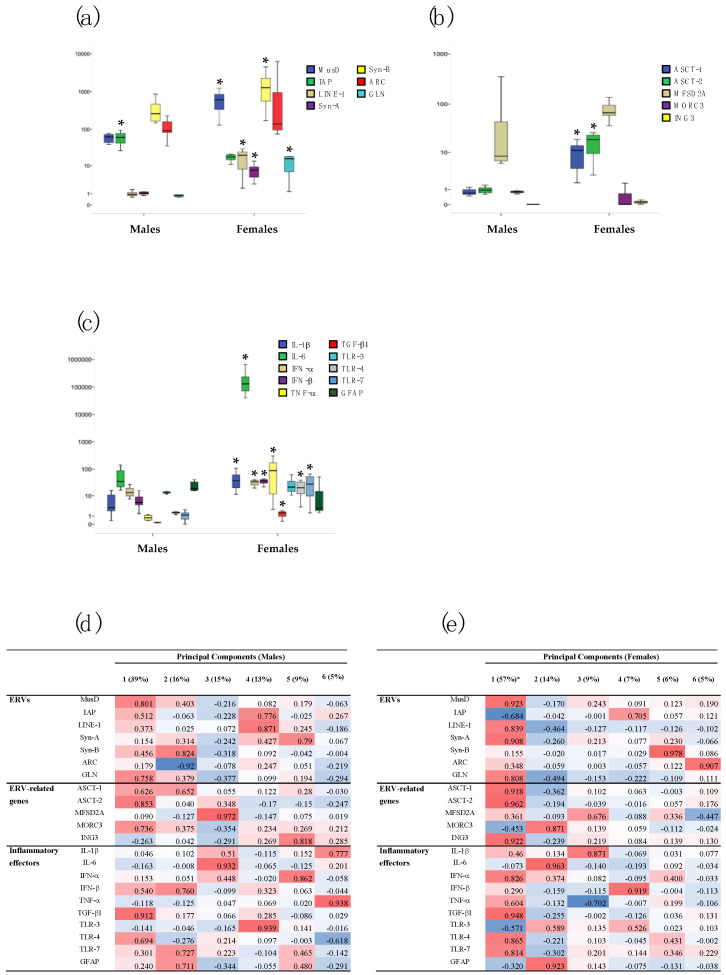
Relative expression of several ERV families (**a**), ERV-related genes (**b**), and inflammatory mediators (**c**) in prefrontal cortex (PFC) of Poly I:C male and female mice. Data are represented as box plots with median value (black horizontal line) and first/third interquartile range. * *p* < 0.05 (males vs. females). PCA and hierarchical clustering of the transcriptional levels of the same genes in PFC samples of Poly I:C male (**d**) and female (**e**) mice. Percentage of total variance explained by the component is reported in the head columns between parentheses; red indicates positive association, blue indicates negative associations, and color intensity refers to association strength.

In the PFC, the expression levels of MusD (*p* = 0.001), LINE-1 (*p* < 0.001), Syn-A (*p* < 0.001), Syn-B (*p* = 0.007), and GLN (*p* < 0.001) were significantly higher in Poly I:C females than in males, while the expression of IAP was higher in males (*p* = 0.010) (Figure 4a). The transcriptional activity of ASCT-1 and ASCT-2 was also higher in females than in males (*p* < 0.001 for all genes) (Figure 4b). Concerning the inflammatory mediators, the expression levels of IL-1β (*p* = 0.001), IL-6 (*p* < 0.001), IFN-α (*p* = 0.001), IFN-β (*p* < 0.001), TNF-α (*p* < 0.001), TGF-β1 (*p* < 0.001), TLR-4 (*p* < 0.001), and TLR-7 (*p* = 0.001) were also significantly higher in female mice than in male mice (Figure 4c). No statistically significant differences were observed between the two sexes in the HP and BL samples (Supplementary Tables S3 and S4). 

PCA performed on PFC samples from Poly I:C males demonstrated that six mediators explain 97% of the total variance (panel d): (i) Component 1, which carried 39% of variance, was positively related to MusD, GLN, ASCT-2, MORC3, and TGF-β1; (ii) Component 2 (~16%) was positively related to Syn-B, IFN-β, TLR-7, and GFAP and negatively loaded on ARC; (iii) Component 3 was positively related to MFSD2A and IL-6 (~15%); and (iv) Component 4 was positively related to IAP, LINE-1, and TLR-3 (~13%). The other two components explained ~9% and ~5% of the variance (respectively) and were positively related to Syn-A, ING3, IFN-α, IL-1β, and TNF-α. The same analysis conducted on the female group also showed that, in this case, six factors explained 98% (panel e); however, the first one carried most of the information: (i) Component 1 (57%) was mainly related to ERVs (with positive loadings on MusD, Syn-A, and GLN and negative loading on LINE-1), ERV-related genes (positive loadings on ASCT1, ASCT2, and ING3) and, to a lesser extent, to inflammatory mediators (positive loadings on IFN-α, TGF-β1, TLR4, and TLR-7) and (ii) Component 2 (14%) was positively related to MORC3, IL-6, and GFAP. The other four components explained ~9%, ~7%, ~6%, and ~5% of the variance (respectively) and were mainly positively related to IL-1β, IAP, IFN-β, Syn-B, and ARC and negatively to TNF-α.

### 2.3. Behavioral Profile of Poly I:C Mice and Correlations of Social Behavioral Responses with ERV, ERV-Related Genes, and Inflammatory Mediators

At variance with Vehicle mice of both sexes and Poly I:C females, Poly I:C males did not exhibit a preference for the social stimulus during the three-chamber test. Poly I:C males spent a similar amount of time sniffing either the cage containing the unfamiliar partner or the inanimate object (prenatal treatment x cage stimulus interaction: F(1,14) = 3.00; social vs. object: *p* < 0.01 (Vehicle males), ns (Poly I:C males); see Supplementary Figure S1a). In contrast, the anxiety profile evaluated in the elevated plus maze and stereotyped responses assessed in marble burying did not reveal any alterations in Poly I:C mice compared to Vehicles (Supplementary Figure S1b,c).

In males (considering both Vehicle and Poly I:C mice), correlation analyses between social sniffing duration and ERV, ERV-related genes and inflammatory mediators in the PFC showed that MFSD, IL6, IFN-α, and TLR-3 were negatively correlated (R= −0.654, *p* = 0.0048, R= −0.773, *p* = 0.0002, R= −0.639, *p* = 0.0064, R= −0.549, *p* = 0.0261, respectively), and TGF-β1 and ING3 were positively correlated (R= 0.541, *p* = 0.0289, R= 0.549, *p* = 0.0262, respectively) with social sniffing. For the HP, significant negative correlations were found between social sniffing and IAP, SYNB, IL6, IFN-α, IFN-β, TLR-3, and GLN (R= −0.507, *p* = 0.0438, R= −0.549, *p* = 0.0260, R= −0.550, *p* = 0.0257, R= −0.571, *p* = 0.0193, R= −0.762, *p* = 0.003, R= −0.682, *p* = 0.0027, R= −0.537, *p* = 0.0378, respectively), whereas ING3 was positively correlated with the same parameter (R= 0.534, *p* = 0.0318). No significant correlation was evident when BL values were evaluated. 

In females, no significant correlations were detected for all markers and matrices assessed.

## 3. Discussion

In this paper, we demonstrated that in C57BL6/J mice, prenatal exposure to Poly I:C induced tissue-specific differential expression of several ERVs, ERV-related genes, and inflammatory mediators. Specifically, both in the prefrontal cortex and hippocampus, the prenatal challenge deregulated the expression of some ERVs and ERV-related genes, while no changes were observed in the blood. Such deregulation across the two considered brain regions (PFC and HP) could also reflect the MIA-induced activation of microglia, the resident immune cells of the brain, which are an important source of key inflammatory mediators [5]. The expression profile found was in line with our previous results from preclinical studies involving the idiopathic BTBR mouse strain and the VPA-induced mouse model of ASD [31,32,33]. In both models, high expression of ERVs was found in whole embryos and brain tissues throughout the lifespan whereas blood expression levels progressively decreased with age. Such differences in ERV expression observed in the two tissues could be attributed to the different cell turnover. In fact, since the cell turnover in the brain is very slow, the increased ERV expression was maintained throughout the lifespan, whereas the rapid turnover that occurs in blood could “dilute” in these cells the effect of the prenatal insult on the ERV transcription, whose levels were higher only at early postnatal stages [31]. 

High expression levels of Syn-B were found in the brain tissues of Poly I:C mice, suggesting its potential role in derailed neurodevelopment and neuroinflammation. This hypothesis is supported by findings in humans in which syncytins play key roles during pregnancy by mediating the fusion of trophoblasts to form syncytiotrophoblast and suppressing maternal immune responses against the fetus [34,35,36,37]. In mice, Syn-A and Syn-B are specifically expressed in the placenta, where the feto-maternal exchanges take place [38,39]. Of note, since the expression of Syn-B has also been detected in primary cultures of hippocampal neurons or cortical glia from C57BL6/J mice treated with Influenza A virus, a possible role of syncytins in mouse brain functions has been suggested [40]. Moreover, the elevated expression of Syn-B in brain tissues parallels the expression of its putative receptor, suggesting that, as already described in humans, MFSD2A may also be related to ERV activity in mice. In fact, several human studies have documented that Syncytin-1 protein interacts with the type D mammalian retrovirus receptor ASCT-1/ASCT-2 on cell membranes, while Syncytin-2 interacts with a different receptor, MFSD2A [41,42,43,44]. 

Another intriguing observation is that the high expression of ERVs and related genes was found in both PFC and HP together with a reduction in ING3 expression, a newly identified ERV transcriptional repressor that seems to prevent innate immune activation in vitro [45]. In this context, ING3 deficiency has been shown to lead to the desuppression of ERV, activation of MDA5-MAVS signaling, and excessive IFN production in HT-29 ING3 KO cells [45]. The mechanism by which ING3 acts could be related to direct binding to DNA sequences and motifs, although definitive evidence that ERV-derived RNAs are recognized by ING3 is still lacking. In HP from Poly I:C mice, MORC3 was highly expressed in parallel with ERV activation despite its function as an epigenetic silencer of transposable elements (TEs) in mouse embryonic stem cells [46]. Thus, MORC3 may not be involved as a “gatekeeper” of ERV expression in the central nervous system. Finally, in the PFC, high levels of ARC were found. ARC, a neuronal gene likely originating from a vertebrate lineage of Ty3/gypsy retrotransposons, is involved in the long-lasting information storage in the mammalian brain and synaptic homeostasis [47]; thus, its deregulation could contribute to the derails of central nervous system development.

In substantial agreement with the present findings, RNA sequencing analysis of brain transcriptomic changes after MIA in a nonhuman primate model has recently provided interesting results concerning the differential expression of TEs and their regulators in several brain areas of MIA-exposed rhesus macaques. Together with the downregulation of PIWIL2 (an inhibitor of the expression of TEs) in the brains of MIA-exposed rhesus macaques, these authors also observed the increased expression of several TEs, including HERVs and long terminal repeat families (e.g., HERV1_LTRa, LTR10E, LTR25, and L1M6) [48].

In addition to the analysis of the expression of ERVs and ERV-related genes in Poly I:C mice, we also investigated the activity of different immune mediators that were induced by the prenatal exposure to the viral mimic. The current findings are in agreement with our previous data on BTBR mice and prenatally VPA-treated mice in which higher expression levels of IL-1β, IL-6, TNF-α, TLR3, and TLR4 have been reported [31] along with the well-documented deregulation of the immune response in Poly I:C model [49]. Inflammation is extensively involved in diverse physio-pathological processes during pregnancy [50]. Indeed, on the one hand, certain inflammatory responses are considered a normal part of different phases comprising immune tolerance and initiation of delivery [51]; on the other hand, an excessive inflammation contributes to the pathogenesis of major diseases of pregnancy and can also be one of the developmental origins of diseases in adulthood [52,53,54]. Finally, the expression of GFAP, known as a marker of CNS damage and neuroinflammation [55] was increased in the PFC from Poly I:C mice, in line with a previous report in preclinical models with an ASD-like phenotype induced by early immune activation [56].

Interestingly, here, we found a potential complex biomarker of neuroinflammation in Poly I:C mice by PCA analysis, comprising ERVs together with IFNs, suggesting a possible relationship among these elements. The interplay among LINE-1, ERVs, and the interferon signaling system has already been hypothesized, suggesting the role of retroviral sequences in shaping of immune systems acting on the IFN network [57,58,59,60]. The mechanisms by which ERVs can shape the innate immune response include the regulation of neighboring gene expression and stimulation of pattern recognition receptors. The upregulation of ERV transcription can lead to the release of ERV-derived pathogen-associated molecular patterns, which stimulate the production of pro-inflammatory mediators [58]. Moreover, the involvement of ERVs in the host antiviral immune system seems to be linked to the IFN pathway by acting as enhancer elements to directly affect the expression of adjacent interferon-stimulated genes [60,61]. Since ERVs are physiologically expressed in humans [23] or can be activated by microenvironmental stimuli (pathogens, drugs, cytokines, etc.), they can provide continuous triggers to host innate immune sensors. On the other hand, inflammatory mediators can further increase ERV activity. Recently, we demonstrated that in vitro exposure of peripheral blood cells from healthy donors to SARS-CoV-2 spike protein induced an early expression of HERV-W, preceding the induction of IL-6, suggesting a role for HERV activation in the inflammation process related to infectious diseases [62].

Thus, in the complex scenario of neurological diseases, the feedback loop made by ERV upregulation and inflammatory mediators could lead to chronic stimulation of the immune system that could sustain the development and/or progression of CNS diseases.

Another main focus of the study was to verify the sex-dependent impact of the prenatal immune challenge on the expression of ERV families, ERV-correlated genes, inflammatory mediators. The results demonstrated that, only in PFC expression levels of some retroviral elements, related genes and inflammatory mediators were higher in females than in males. In this regard, the PCA revealed that in females, the variance was accounted for mainly from ERVs and ERV-related genes, whereas this pattern was not so evident in males. A whole PCA analysis illustrated that ERV, ERV-related gene, and immune mediator expression levels are coherently well-orchestrated in the prefrontal cortex, less coherently but still orchestrated in the hippocampus, and totally unrelated in blood. Moreover, when PCA on prefrontal markers was performed for the two sexes separately, the female PCA pattern was the clearest, with the first component featuring mostly positive scores in ERV, ERV-related genes, and immune mediators and the second one featuring mostly negative scores. This picture was more similar to the one reported in the PCA from both sexes pooled. The larger effects of Poly I:C in females were in agreement with our previous findings, demonstrating a more marked influence on ERV activity and on somatic and motor development in females prenatally exposed to VPA [33]. It is noteworthy that that the sample size (max. 8 in each experimental group) was not fully adequate to identify behavioral alterations; a clear social deficit was evident in Poly I:C males within the present dataset. Importantly, social response was significantly correlated with selected inflammatory markers and ERV-related genes in the PFC and HP, two brain regions with crucial roles in the control of social behavior [63].

A sex-linked molecular signature comprising ERVs and immune mediators has also been proposed in ASD families, in which only ASD children and their mothers share high levels of expression of some human ERVs and cytokines in peripheral cells, whereas no association was found with fathers. The common expression profile in ASD children and their mothers, and the discrepancy with fathers, support the hypothesis of maternal imprinting as a contributing factor in increasing susceptibility to neurodevelopmental disorders [64,65]. As such, the elevated ERV transcriptional activity in females could be due to the combination of a variety of complex female physiological events (e.g., oogenesis, fertilization, fetal stem cell development, placentation, and pregnancy maintenance) that provide selective pressures on ERV activity [66]. Although molecular mechanisms underpinning sex differences in gene regulation are currently unknown, it is likely that genes linked to sex chromosomes, hormonal changes, and/or their interactions may be involved [67,68,69,70]. In addition, sex-related differences in neuroinflammatory responses (e.g., upregulation of anti-inflammatory markers only in females) induced by perinatal immune activation raises the possibility of protective or resilience-related processes that reduce ASD prevalence in females [56]. 

## 4. Materials and Methods

### 4.1. Animals and Treatments

C57BL6/J mice (Jackson Laboratory, Bar Harbour, ME, USA) were housed under standard housing conditions (temperature 21 ± 1 °C and relative humidity 60 ± 10%) with food and water ad libitum, under a 12:12 reverse light cycle (lights on at 6:00 P.M.). Two weeks after their arrival, mice were mated (2 females to 1 male) and females were checked twice a day for the presence of the vaginal plug, noted as gestational day (GD) 0.

At GD 12.5, pregnant female mice were weighed and received a single injection of Poly I:C (potassium salt; Sigma-Aldrich, #P9582; 20 mg/kg, i.p.) or Vehicle (Veh, 0.9% NaCl). All pups remained with their mother until postnatal day (PND) 28, when they were weaned and housed with same-sex littermates (2–3 mice per cage). 

### 4.2. Behavioral Assessment

From PND 35 to PND 60, mice of both sexes were assessed for the anxiety profile, repetitive or stereotyped behaviors, and preference for social stimuli (see Supplementary Materials). 

### 4.3. Tissue Collection

At the end of behavioral testing (PND 60), mice of both sexes (Poly IC: 8 males and 9 females; Veh: 8 males and 6 females) were sacrificed and brain (prefrontal cortex (PFC) and hippocampus (HP)) and blood (BL) samples were collected and stored at −80 °C until use. 

### 4.4. RNA Extraction from PFC, HP, and BL Samples

RNA isolation from PFC and HP samples (right cerebral hemisphere) was performed using a Total RNA purification kit (Norgen Biotek Corp, Thorold, Canada) according to the manufacturer’s instructions, starting from 10 mg or less of tissue. Briefly, after adding the lysis buffer, samples were homogenized using the plunger of a syringe and by passing through a syringe needle. Samples were filtered through a 100-mesh nylon textile, mixed with 70% ethanol, and transferred to an RNA mini spin column. Treatment with RNase-free DNase (Promega, Italy) was performed “in column” at room temperature for 15 min to ensure removal of contaminating DNA. Finally, RNA was eluted in 50 μL of RNAse-free water. 

Total RNA isolation from BL samples was performed, starting from 200 μL of BL, by using TRIzolTM (Invitrogen, MA USA), according to the manufacturer’s instructions. When the sample volume was less than 200 μL, the amount was achieved by adding phosphate-buffered saline (PAN-Biotech, Aidenbach, Bavaria). Contaminating DNA was removed by a DNase treatment for 15 min at room temperature and RNA was resuspended in 40 μL of RNase-free water. 

RNA of all samples included in the study was evaluated by Nanodrop DS 11 (DeNovix, DE, USA), showing a 260/280 ratio of approximately 2.0 and a concentration ranging from 30 to 200 ng/μL. 

### 4.5. RT Real-Time PCR Assay

RNA obtained from PFC, HP, and BL samples was reverse-transcribed into cDNA using the Improm-II Reverse Transcription System (Promega, Fitchburg, Wisconsin, USA) according to the manufacturer’s protocol. For the reaction, 200 ng of RNA obtained from PFC, HP, and BL samples was used for the retrotranscription and cDNA samples were diluted (1:10). 

The transcriptional levels of eight ERV families, such as mouse type D retrovirus (MusD), Intracisternal A particle (IAP), Long Interspersed Nuclear Element-1 (LINE-1), Syncytin 1 and 2 (Syn-A, Syn-B), Activity Regulated Cytoskeleton-Associated Protein (ARC), and murine retrovirus using tRNAGln (GLN); five ERV-related genes, such as MORC family CW-type zinc finger 3 (MORC3), inhibitor of growth family member 3 (ING3), Alanine-/Serine-/Cysteine-/Threonine-preferring Transporter (ASCT-1, ASCT-2), and major facilitator superfamily domain containing 2A (MFSD2A); cytokines, such as interleukin-1β (IL-1β), IL-6, interferon (IFN-α, IFN-β), tumor necrosis factors α (TNF-α), and transforming growth factor β1 (TGF-β1), toll-like receptors (TLR-3, TLR-4, and TLR-7); and a marker of neuroinflammation, the glial fibrillary acidic protein (GFAP), were quantitatively assessed by quantitative RT real-time PCR assay. The assays were performed in a Bio-rad instrument (CFX96 Real-Time System, Bio-rad, Hercules, California, USA) using SYBR Green chemistry (SensiFAST™ SYBR, NO-Rox Kit, Meridian Bioscience, USA) with specific primer pairs, and are listed in Table 1. To set-up the real-time reaction, a (10-fold) serial dilution was performed to calculate efficiencies and correlation coefficient, by the formula efficiency = 10 (−1/slope) and all primer pairs used showed an efficiency ranging from 0.96 to 0.97. The RT real-time PCR reaction included 2 μL of cDNA, 10 μL of SensiFAST™ SYBR Mix, and forward and reverse primers ranging from 100 to 200 nM in a total volume of 20 μL. Forward and reverse primers were used at a specific concentration, as follows: MusD (accession no AC102360), IAP (accession no AB099818.1), LINE-1 (accession no NM_001081202.1), GLN (accession no JF714652), ARC (accession no NM_173420.3), MORC3 (accession noNM_001045529.3), ING3 (accession no NM_001311061.1), ASCT-1 (accession no NM_018861.3), ASCT-2 (accession no NM_009201.2), IFN-α (accession no NM_010502.2), IFN-β (accession no NM_010510.1), IL-6 (accession no NM_001314054.1), TNF-α (accession no NM_001278601.1) (200 nM), Syn-A (accession no NM_001013751.2), Syn-B (accession no NM_173420.3), MFSD2A (accession no NM_029662.2), IL-1β (accession noNM_008361.4), TLR-7 (accession no NM_001290755.1), GFAP (accession no NM_010277.3), GAPDH (accession no NM_001289726.1) (100 nM), TGF-β1 (accession no NM_011577.2), TLR-3 (accession no NM_001357316.1), and TLR-4 (accession no NM_021297.3) (300 nM).

The reaction was conducted for 1 cycle at 95 °C for 3 min, for 40 cycles at 95 °C for 45 s, and at 60 °C for 1 min. Each sample was analyzed in triplicate and a negative control (no template reaction) was included in each experiment to determine any possible contamination. The housekeeping glyceraldehyde 3-phosphate dehydrogenase gene (GAPDH) was used to normalize the results. Each experiment was completed with a melting curve analysis to confirm the specificity of amplification and the lack of any nonspecific product and primer dimer. Quantification was performed by the comparative threshold cycle (Ct) method. The relative expression of C57BL6/J mice treated with Poly I:C compared with Vehicle mice was calculated as follows: 2^−[ΔCt (sample)—ΔCt (calibrator)]^ = 2^−ΔΔCt^. The comparison of GAPDH Cts among the groups did not show significant differences.

### 4.6. Statistical Analysis

Statistical analysis of groupwise expression levels was performed through a nonparametric Mann–Whitney test to compare the ERVs’, ERV-correlated genes’, inflammatory cytokines’, and TLRs’ transcriptional levels, in PFC, HP, and BL samples obtained from C57BL6/J offspring at PND 60, in utero, treated with Poly I:C or Vehicle. 

To identify associations between biomarkers in a multivariate manner, we performed a principal component analysis (PCA) followed by varimax rotation and Kaiser normalization. Factors were retained when associated with eigenvalues larger than one and loadings were extracted through regression methods. The PCA was repeated separately in different groups, Vehicle and Poly I:C mice, and among Poly I:C mice, in males and females. 

Behavioral data were analyzed by two-way analysis of variance (ANOVA) with prenatal administration (Veh or Poly I:C) and sex as between factors (with repeated measures with stimulus being within factors for three-chamber social test) followed by Tukey’s post hoc test on significant interaction effects. Pearson correlations were applied to associate behavioral responses with ERVs, ERV-related genes, and inflammatory markers within each sex. Statistically significant comparisons were considered at *p* < 0.05. Data analyses were performed using the SPSS statistical software system (version 24.0 for Windows, IBM Corp., Armonk, NY, USA).

## 5. Conclusions

To conclude, these results (i) designate ERV activation as a common feature shared by several risk factors for ASD, (ii) suggest ERVs as biomarkers of changes occurring in the brain of Poly I:C mice, primarily in female offspring, and (iii) reinforce the view of the differential vulnerability of the two sexes to ASD risk. As such, a deep characterization of the molecular mechanisms by which sex differences affect neurodevelopment in preclinical models, will help in identifying gender-specific diagnosis and personalized treatment strategies. Importantly, the present findings support further investigations aimed at (i) verifying whether pharmacological modulation of ERV activity and inflammation has an impact on the neurobehavioral profile in ASD animal models (involving a larger number of subjects) and (ii) transcriptomic profiling to obtain a more detailed picture of the ERV and immune expression patterns. It would also be relevant to investigate whether in vitro exposure to pathogen-derived antigens in murine neuronal cell culture may modulate ERVs and immune mediators to figure out the cause and effect of this complex interplay.

## Figures and Tables

**Table 1:** Specific primer pairs for the RT real-time PCR assay.

Gene		Primers Pairs (5′ to 3′)	Ref.
MusD	Forward	GTTAAACCCGAGCGCTGGTTC	[71]
	Reverse	GCTATAAGGCCCAGAGAGAAATTTC	
IAP	Forward	AAGCAGCAATCACCCACTTTG G	[71]
	Reverse	CAATCATTAGATGYGGCTGCCAAG	
LINE-1 ORF2 *	Forward	TGCAGAATTGACAAATGGGA	
	Reverse	ATCCTTTCCCAGTCTGTTGG	
Syn-A *	Forward	AGAGCCATGGTTCGTCCTTG	
	Reverse	CCAAGTCCTTAGTGGGGCTG	
Syn-B *	Forward	ACATTGAAAGACGCCTCCGT	
	Reverse	CGCCCTTCTGTCAGGATTGT	
ARC	Forward	GAGAGCTGAAAGGGTTGCAC	[72]
	Reverse	GCCTTGATGGACTTCTTCCA	
GLN *	Forward	ATCACCCTGCATCCAGTTTAG	
	Reverse	TATTGCCGCTAGGTCTTCATT	
ASCT-1 *	Forward	TATGCTGGGCCATGTCATCC	
	Reverse	GGAACAGGTCGCAAAAGCTG	
ASCT-2 *	Forward	GCCTGGTGGTCTTCGCTATC	
	Reverse	GGAACAGGATTCCAACGGGT	
MFSD2A *	Forward	CTCCTGGCCATCATGCTCTC	
	Reverse	GGCCACCAAGATGAGAAA	
MORC3	Forward	TGTGAAGAGCTGCAGACTGA	
	Reverse	ATCAGGCACGATCATAGCCA	
ING3	Forward	TTCACATACTCCCGTGGAAAA	[73]
	Reverse	GCGCTTCAGATTTGAATTTCTT	
IL-1β	Forward	TTGACGGACCCCAAAAGATG	[74]
	Reverse	AGAAGGTGCTCATGTCCTCA	
IL-6	Forward	GTTCTCTGGGAAATCGTGGA	[74]
	Reverse	TGTACTCCAGGTAGCTATGG	
IFN-α *	Forward	TGCAGGAATTTCCCCTGACC	
	Reverse	GGCTCTCCAGACTTCTGCTC	
IFN-β	Forward	CGTGGGAGATGTCCTCAACT	[75]
	Reverse	AGATCTCTGCTCGGACCAACC	
TNF-α *	Forward	ATCGGTCCCCAAAGGGATGA	
	Reverse	TCCACTTGGTGGTTTGTGAGTG	
TGF-β1	Forward	TGACGTCACTGGAGTTGTACGG	[74]
	Reverse	GGTTCATGTCATGGATGGTGC	
TLR-3 *	Forward	CCAGGCTCTGGAAACGCGCA	
	Reverse	ATGTGGAGGTGAGACAGCCCC	
TLR-4 *	Forward	AGATCTGAGCTTCAACCCCTTG	
	Reverse	ATTGTTTCAATTTCACACCTGGA	
TLR-7 *	Forward	TGGCTCCCTTCTCAGGATGA	
	Reverse	ATGTCTCTTGCTGCCCCAAA	
GFAP *	Forward	CAGATCCGAGAAACCAGCCT	
	Reverse	ACACCTCACATCACCACGTC	
GAPDH	Forward	AACGACCCCTTCATTGAC	[71]
	Reverse	CTCCACGACATACTCAGCAC	

* Primers designed by NCBI Primer-BLAST.

## Data Availability

The data presented in this study are available on request from the corresponding author.

## Supplementary Materials

The following supporting information can be downloaded at: https://www.mdpi.com/article/10.3390/ijms232213930/s1, Supplementary Materials and Methods: Behavioral Assessment; Supplementary Table S1: Median value, interquartile range (IQR), and *p*-values of the expression levels of ERVs, ERV-related genes, and inflammatory mediators in prefrontal cortex, hippocampus, and blood samples from Vehicle-treated mice and Poly I:C-treated mice; Supplementary Table S2: Median value, interquartile range (IQR), and *p*-values of the expression levels of ERVs, ERV-related genes, and inflammatory mediators in prefrontal cortex from males and females prenatally exposed to Poly I:C; Supplementary Table S3: Median value, interquartile range (IQR), and *p*-values of the expression levels of ERVs, ERV-related genes, and inflammatory mediators in hippocampus from males and females prenatally exposed to Poly I:C; Supplementary Table S4: Median value, interquartile range (IQR), and *p*-values of the expression levels of ERVs, ERV-related genes, and inflammatory mediators in blood samples from males and females prenatally exposed to Poly I:C. Supplementary Figure S1: Behavioral responses in juvenile/adult offspring. Poly I:C administration induced (**a**) deficits in preference for a social stimulus but it did not affect anxiety-like profile (**b**) and repetitive behaviors (**c**).
