# Supplementary material for: Differential Expression of Endogenous Retroviruses and Inflammatory Mediators in Female and Male Offspring in a Mouse Model of Maternal Immune Activation
Source: Int J Mol Sci. 2022 Nov 11;23(22):13930. doi: 10.3390/ijms232213930 (PMC9695919; doi:10.3390/ijms232213930)
Supplement: Supplementary file 1 [file ijms-23-13930-s001.zip › Supplementary Materials and methods.pdf]

Supplementary Materials and Methods

Behavioural testing

Three-Chamber social test

To assess the preference for social stimuli, mice were tested in the three-chamber social test. The apparatus was a Plexiglas box (60 × 40 cm) divided into three chambers connected by doorways. The subject mouse was acclimated to the empty apparatus for 10 min before the sociability test and then confined to the center chamber. An object enclosed in an inverted wire cup was introduced into one of the side chambers while an unfamiliar, age- and sex-matched mouse was placed under an identical wire cup in the other side chamber. The subject was then allowed to access to all three chambers for 10 min. Side chamber location of the object and the social stimulus were counterbalanced across subjects. The time spent in each chamber and the time spent sniffing each cup were recorded and analyzed by The Observer XT-15 software (Noldus, Wageningen, The Netherlands).

Elevated Plus Maze

To assess the anxiety-like behavior, mice were tested in the EPM based on the conflict between the exploration of new areas and avoidance of unsafe areas. The EPM was a Plexiglas cross-shaped maze, 60 cm high above the floor, consisting of two open and two closed arms. Each mouse was placed in the center of the maze facing an open arm and allowed to explore the maze for 5 min. Frequencies of total, open and closed entries (all four paws into an arm) and time spent in each arm were analyzed by The Observer XT-15 software (Noldus, Wageningen, The Netherlands).

Marble Burying test

To assess repetitive/stereotyped behaviors, mice were tested in the Marble Burying test. Twenty navy blue glass marbles (1.2 cm in diameter) were arranged in a symmetrical 4 × 5 matrix on top of 3 cm deep bedding in a clean cage (36 × 20.5 × 18.5 cm). Each mouse was placed in the center of the cage and allowed to explore the cage for a 30 min test period. A marble was considered buried when it was covered by bedding more than 50%. The number of marble buried was scored manually by a trained investigator blinded to experimental group.
