# Supplementary figures and images for: Differential Expression of Endogenous Retroviruses and Inflammatory Mediators in Female and Male Offspring in a Mouse Model of Maternal Immune Activation
Source: Int J Mol Sci. 2022 Nov 11;23(22):13930. doi: 10.3390/ijms232213930 (PMC9695919; doi:10.3390/ijms232213930)

**(a) Three Chamber social test**

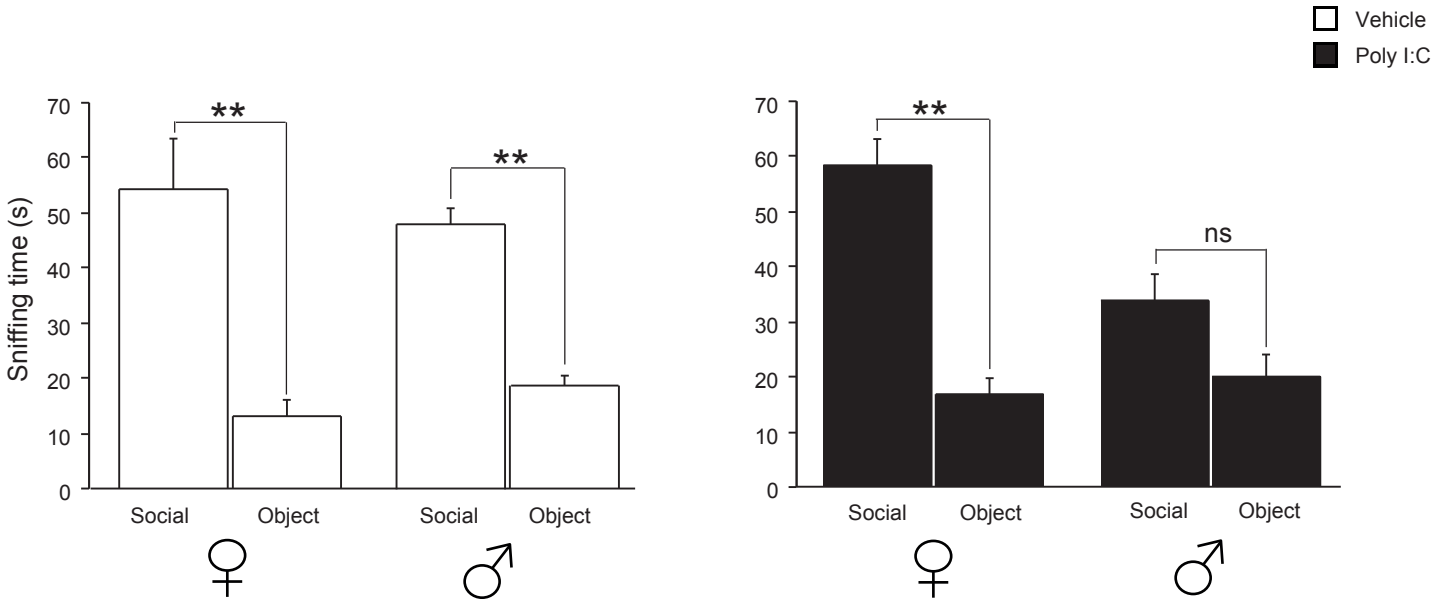

**(b) Elevated Plus Maze test**

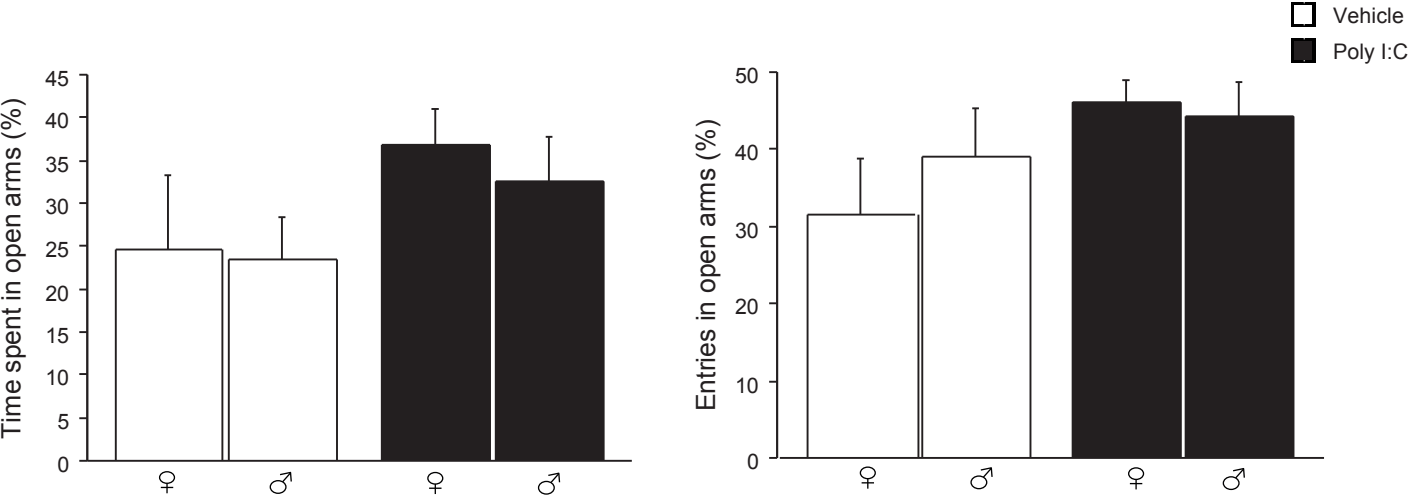

**(c) Murble Burying test**

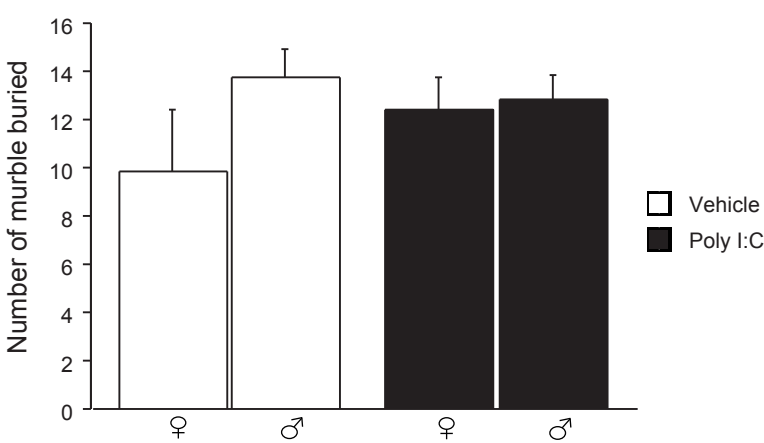

Supplement: Supplementary file 1 [file ijms-23-13930-s001.zip › Supplementary Figure S1.pdf]
